# Associative cued asynchronous BCI induces cortical plasticity in stroke patients

**DOI:** 10.1002/acn3.51551

**Published:** 2022-04-30

**Authors:** Imran Khan Niazi, Muhammad Samran Navid, Usman Rashid, Imran Amjad, Sharon Olsen, Heidi Haavik, Gemma Alder, Nitika Kumari, Nada Signal, Denise Taylor, Dario Farina, Mads Jochumsen

**Affiliations:** ^1^ Health and Rehabilitation Research Institute and BioDesign Lab Auckland University of Technology Auckland New Zealand; ^2^ SMI, Department of Health Science and Technology Aalborg University Aalborg Denmark; ^3^ Centre for Chiropractic Research New Zealand College of Chiropractic Auckland New Zealand; ^4^ Riphah International University Islamabad Pakistan; ^5^ Department of Bioengineering Imperial College London London UK

## Abstract

**Objective:**

We propose a novel cue‐based asynchronous brain–computer interface(BCI) for neuromodulation via the pairing of endogenous motor cortical activity with the activation of somatosensory pathways.

**Methods:**

The proposed BCI detects the intention to move from single‐trial EEG signals in real time, but, contrary to classic asynchronous‐BCI systems, the detection occurs only during time intervals when the patient is cued to move. This cue‐based asynchronous‐BCI was compared with two traditional BCI modes (asynchronous‐BCI and offline synchronous‐BCI) and a control intervention in chronic stroke patients. The patients performed ankle dorsiflexion movements of the paretic limb in each intervention while their brain signals were recorded. BCI interventions decoded the movement attempt and activated afferent pathways via electrical stimulation. Corticomotor excitability was assessed using motor‐evoked potentials in the tibialis‐anterior muscle induced by transcranial magnetic stimulation before, immediately after, and 30 min after the intervention.

**Results:**

The proposed cue‐based asynchronous‐BCI had significantly fewer false positives/min and false positives/true positives (%) as compared to the previously developed asynchronous‐BCI. Linear‐mixed‐models showed that motor‐evoked potential amplitudes increased following all BCI modes immediately after the intervention compared to the control condition (*p* <0.05). The proposed cue‐based asynchronous‐BCI resulted in the largest relative increase in peak‐to‐peak motor‐evoked potential amplitudes(141% ± 33%) among all interventions and sustained it for 30 min(111% ± 33%).

**Interpretation:**

These findings prove the high performance of a newly proposed cue‐based asynchronous‐BCI intervention. In this paradigm, individuals receive precise instructions (cue) to promote engagement, while the timing of brain activity is accurately detected to establish a precise association with the delivery of sensory input for plasticity induction.

## Introduction

Stroke is one of the leading causes of acquired disability among adults.^1^ Of those who survive a stroke, up to 80% suffer from motor impairments,^2^, ^3^ and up to 50% require assistance with daily activities.^4^, ^5^, ^6^ This ongoing disability is partly due to the difficulty in finding effective rehabilitation approaches for this heterogeneous injury.^7^

Recent rehabilitation strategies are based on the principles of motor learning^8^ and the associated neural plasticity.^9^ Brain–computer interfacing (BCI) has been used to pair electroencephalographic (EEG) signals associated with attempted hemiparetic limb movements, with the activation of somatosensory pathways via peripheral electrical stimulation or robotic‐assisted passive movement.^10^ This BCI approach induces neural plasticity, as evidenced by an increase in corticomotor excitability,^11^, ^12^, ^13^, ^14^ and has been shown to improve upper and lower extremity functions in individuals with stroke,^11^, ^15^, ^16^, ^17^, ^18^ suggesting it can facilitate motor learning.^9^ The effect is hypothesized to occur through Hebbian associative plasticity because depends on precise timing between the detection of movement intention and the arrival of somatosensory afferent volleys in the brain.

Associative‐BCIs require the early detection of movement intention to provide a precise association with afferent volleys.^19^ The detection of movement intention can occur within a synchronous cue‐based BCI system, where the EEG analysis is synchronized with periods when the individual is cued to move, or within an asynchronous self‐paced BCI system, where the EEG is analyzed continuously while the individual performs self‐paced movements.^11^, ^12^, ^13^, ^14^ We will refer to the first approach as offline‐BCI since the BCI does not decode the EEG during the intervention. Both offline and asynchronous self‐paced BCIs have been shown to increase cortical excitability. The asynchronous self‐paced BCI provides slightly superior effects compared to the offline‐BCI in healthy adults,^20^ however, it produces more false‐positive detections because the signal detector is continuously active and prone to falsely detecting movement attempts. These false‐positive detections trigger afferent stimulation, which is then not paired with a motor command. False‐positive detections are avoided within the offline‐BCI (cue‐based), which has the additional advantage of avoiding daily calibration because the BCI system is offline (i.e., disconnected) during the intervention phase.^21^ In addition, the offline‐BCI may be more engaging because movements are cued. However, the offline‐BCI triggers afferent stimulation with each cue, regardless of the patient's compliance, and does not account for between‐trial variability in movement timing, which may result in less precise timing of afferent feedback.

Here we propose a novel BCI intervention that combines a cue‐based paradigm with the asynchronous self‐paced BCI. The core concept is that the individual is cued to attempt a movement but the BCI only decodes the timing of movement intention within the time intervals of the cue. It was hypothesized that the number of false‐positive detections would be reduced by operating the BCI in the proposed cue‐based asynchronous mode where the user only attempt movements in pre‐determined time windows, and the detector is disabled outside these windows. This study investigates the effect of the proposed cue‐based asynchronous‐BCI on corticomotor excitability, compared to the offline BCI, asynchronous‐BCI, and a control intervention, in individuals with stroke.

## Materials and Methods

The study employed a repeated measures crossover design made up of three active interventions and one control intervention. All procedures were approved by the local ethics committee (Riphah/RCRS/REC/00483). The participants provided their informed consent prior to participation. All procedures were performed according to the declaration of Helsinki.

### Participants

Twelve patients with stroke (see Table 1) were recruited from Railway General Hospital, Rawalpindi affiliated with Riphah International University, Pakistan. The inclusion criteria were that the patients (i) had impaired ankle dorsiflexion movement following a stroke, and were able to (ii) provide informed consent and follow instructions, (iii) move index finger of both hands (discernable with eyes), (iv) tolerate transcranial magnetic stimulation (TMS), and (v) had motor evoked potentials (MEPs) in response to the TMS for the paretic tibialis anterior (TA) muscle at rest.

**Table 1:** Patient demographics.

Patient	Gender	Age (years)	Time since injury (months)	Hemiplegia	Type of stroke
1	M	56	15	Left	Ischemia
2	M	49	29	Right	Ischemia
3	M	36	64	Right	Hemorrhage
4	M	49	18	Right	Hemorrhage
5	M	47	31	Left	Ischemia
6^1^	M	51	23	Left	Ischemia
7	F	53	56	Left	Ischemia
8	M	62	17	Left	Ischemia
9	M	65	12	Left	Ischemia
10^1^	F	48	16	Left	Ischemia
11^1^	F	66	38	Left	Ischemia
12	M	41	27	Left	Hemorrhage

^1^ Patients excluded.

### Experimental procedure

**Figure 1:**
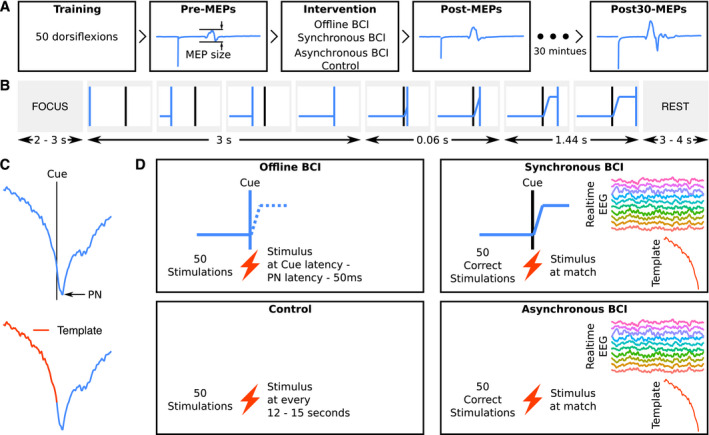
Overview of the experimental procedures. (A) Flow of experiment: TMS was used to elicit 12 MEPs at each pre‐, post‐ and post30‐intervention timepoints. The order of BCI interventions was randomized. (B) The cue shown to participants during the training and intervention (offline and cue‐based asynchronous‐BCIs) phases. The vertical blue line moved from left to right and participants were instructed to dorsiflex their affected side's ankle when the blue vertical line touched the fixed black vertical line. During the intervention (testing) session, for asynchronous‐BCI participants were looking at status of the detector active (green) or inactive (red) continuously, whereas in cue‐based asynchronous‐BCI participants were looking at the visual cue. (C) MRCPs from a representative participant. In the top, a MRCP aligned with the visual cue and PN to be used in the offline‐BCI intervention is shown; in the bottom the template used for cue‐based asynchronous and asynchronous‐BCIs is shown. (D) The four interventions with the presence/absence of a cue and protocol for peripheral nerve stimulations. Abbreviations: Async = asynchronous; PN = peak negativity.

The experiment consisted of four interventions: (1) offline BCI, (2) asynchronous‐BCI, (3) cue‐based asynchronous‐BCI and (4) control. The order of the interventions was randomized, and any two sessions were separated by at least 24 h. At the beginning of each intervention, for training purposes and calibration of the offline BCI, the participants completed 50 attempted dorsiflexion movements of the affected ankle joint while EEG was recorded. These 50 training repetitions were completed in time with a visual cue, and the same cue was used in the subsequent cue‐based asynchronous‐BCI and offline BCI interventions. The asynchronous‐BCI and control interventions did not use the visual cue. The visual cue was displayed on a monitor and had four phases which prompted the participants to (i) bring their attention to the screen (1–2 sec), (ii) prepare for movement (1.5–2 sec), (iii) dorsiflex their ankle (1.5 sec), and then (iv) rest (3–4 sec). The ankle movement was ballistic and against gravity but without resistance. Following the training/calibration session, pre‐intervention MEPs were recorded, and then the BCI intervention was delivered in which 50 peripheral electrical stimuli were paired with the movement attempt. Post‐intervention MEPs were recorded immediately after, and 30 min following the intervention. The overall study flow chart is shown in Figure 1.

### Measurements and stimulation

#### EEG

Continuous EEG was recorded using a 64‐channel REFA amplifier (TMSi, Twente, The Netherlands) with a sampling frequency of 2048 Hz. EEG signals from the following electrodes were used for BCI operation: F3, Fz, F4, C3, Cz, C4, P3, Pz, and P4. These were referenced to the mastoid ipsilateral to the affected limb. Electro‐oculography was recorded from FP1. The impedance of the electrodes was kept below 10 kΩ. The participants were instructed to minimize eye movements and activity in facial muscles and focus on the task.

#### Motor‐evoked potentials

MEPs were recorded from the electromyography (EMG) of TA muscle of the affected limb. Two electrodes (20 mm Blue Sensor Ag‐AgCl, AMBU A/S, Ballerup, Denmark) were placed on the belly of the muscle in a bipolar configuration and the ground electrode was placed on the distal part of the tibia. The EMG was recorded at 4000 Hz with a gain of 1000 (OT Bioelectronica, Turin, Italy). The MEPs were elicited using single‐pulse TMS (Magstim Company, Dyfed, UK) delivered with a figure‐of‐eight double cone‐coil placed in a posterior–anterior current direction. Initially, the optimal stimulation site and resting threshold (RTh) were determined. The optimal stimulation site was defined as the area where the largest MEPs were recorded compared to adjacent areas. The position of the coil was marked on the participant head to ensure the testing position was identical throughout the experiment. RTh was defined as the lowest stimulator output where 5 out of 10 MEPs exceeded 50 *μ*V. Twelve stimuli were delivered at 120% RTh before, immediately after and 30‐min after the interventions. Each stimulus was separated by 5–7 sec.

#### Peripheral electrical nerve stimulation

Electrical stimulation was delivered to the deep branch of the common peroneal nerve of the affected side using two stimulation electrodes (32 mm, PALS, Platinum, Patented Conductive Neurostimulation Electrodes, Axelgaard Manufacturing Co., Ltd., USA) using Digitimer Stimulator DS7AH (Hertfordshire, UK). The cathode was placed proximally, and the anode was placed distally. The stimulation electrodes were placed in such a way that there was activation of the TA muscle without activation of peroneal muscles. The lowest intensity needed to elicit a muscle twitch response in the TA tendon (motor threshold) was identified through palpation. During delivery of the BCI intervention, electrical stimulation was delivered at a stimulus intensity of 110% of the motor threshold with a pulse width of 1 msec.

#### Brain–computer interface systems

The three BCI systems were calibrated with 50 cue‐based movements at the start of their respective sessions. The EEG data from this calibration period were band‐pass filtered from 0.05–10 Hz with a 2nd‐order zero‐phase shift Butterworth filter and downsampled to 32 Hz. For the asynchronous and the cue‐based asynchronous‐BCIs, an optimized spatial filter with Cz as the central electrode was used to obtain a single virtual channel.^13^ For the offline BCI, a Laplacian filter^11^ was used instead with Cz as the central channel. The virtual channels for all three BCI modes were averaged across the trials to obtain an averaged MRCP. The timing of electrical stimulation for each BCI mode is briefly described below but greater detail can be found in^11^ for the offline system and^13^ for the asynchronous system.

##### Offline BCI

The peak negativity of the averaged MRCP was identified. The latency between the peak negativity of the averaged MRCP and the visual cue to attempt the movement was calculated. During intervention delivery, the electrical stimulation was delivered with the timing equal to “visual cue latency – latency of maximum negativity – 50 msec” (see^11^ for details) in each trial for 50 trials in total. The 50 msec were subtracted to account for the conduction time of the afferent stimulus and central processing delays in the brain.^11^

##### Asynchronous‐BCI

From the averaged MRCP, a signal template of the initial negative phase with respect to the onset of the movement (as determined by the cue) was extracted. This EEG template was used to individualize the asynchronous‐BCI system as described in Niazi et al.^13^ During the intervention phase, self‐paced movement attempts were detected from the EEG using a matched filter. For each participant, the threshold for the detector was determined from a receiver operating characteristics curve to optimize the trade‐off between the number of false positive detections and the number of true positive detections. The system was disabled for 5 sec after the system detected a movement intention to ensure a 5–7 sec rest between attempts. The active (green box) or inactive (red box) status of the detector was shown to the participants on the computer screen continuously during intervention (testing) session. Furthermore, if the activity in FP1 exceeded 125 *μ*V the detector was disabled.

##### Cue‐based asynchronous‐BCI

As done in the asynchronous system, a signal template of the initial negative phase with respect to the onset of the movement was extracted from the averaged MRCP. In the cue‐based asynchronous‐BCI, participants were cued to perform attempted movements. However, rather than delivering the electrical stimulation at a pre‐determined time as in the offline system, the stimulation was delivered by detecting the movement attempt online using a matched filter approach similar to the asynchronous system as explained in the above section. The detector was disabled after detection, but the cue remained active. The detector was also deactivated when the activity in FP1 exceeded 125 *μ*V therefore, no stimulation was delivered to the patients.

##### Control intervention

During the control session, the participants received 50 electrical stimulations every 12–15 sec.

##### Validation of the asynchronous and Cue‐based asynchronous systems

During the delivery phase for the asynchronous and cue‐based asynchronous interventions, the detector was deactivated after the electrical stimulation was delivered to the common peroneal nerve. The participants conveyed to the experimenter by extending their right index finger if it was a true positive detection. Otherwise, all the detections were considered false positives (observed by the experimenter from detector status). For false negatives, participants extended their left index finger during the rest phase of the cue‐based asynchronous mode and after approximately 2 sec of not receiving any stimulation during asynchronous intervention. The performance of the system was evaluated using the true positive rate (TPR), number of false‐positive detections per minute (FP/min) and false positive/true positives (%).

#### Data analysis and statistics

The primary null hypothesis for the analysis was that there is no change in the MEP amplitudes from pre‐ to post‐ and post30‐intervention time points in any of the sessions. The secondary null hypothesis was that there is no difference between the control and the BCI sessions at either of the two post‐intervention time points. Peak‐peak amplitudes were extracted from individual MEPs using Signal Software version 4 (CED, UK) and collated in Excel (Microsoft Corporation, USA). The average of 12 MEPs was computed for each participant at each time point (pre‐, post‐ and post30‐) and exported to R version 3.6.3 (R Core Team, Vienna, Austria) for further analysis.

Two separate analyses were conducted to evaluate the absolute and relative differences in MEP sizes across the four intervention sessions (offline BCI, asynchronous‐BCI, cue‐based asynchronous‐BCI, and control) at two time points (post and post30), while accounting for pre‐intervention differences. The analysis in absolute units considered averaged peak‐peak MEP amplitude (MEPabs), whereas the analysis in relative units considered the percentage change in averaged peak‐peak MEP amplitude (MEP%) defined as $\left({\mathrm{MEP}}_{\mathrm{abs}}^{\text{post}}-{\mathrm{MEP}}_{\mathrm{abs}}^{\mathrm{pre}}\right)/{\mathrm{MEP}}_{\mathrm{abs}}^{\mathrm{pre}}\times 100$. Two mixed models were setup in R using packages lme4^22^ version 1.1–21 and *robustlmm* ^23^ version 2.3.

The first linear mixed model regressed the MEPabs on session, time, interaction of session and time, and the averaged peak‐peak MEPs before the intervention. The session, time, and their interaction were entered as discrete variables, whereas averaged peak‐peak MEP amplitude was treated as a continuous variable. The model also estimated a random intercept for each participant to account for the between participant variance in the data.

The second robust linear mixed model regressed the MEP% on session, time and the interaction of session and time. It also estimated a random intercept for each participant. The model was setup using a robust framework as the relative data were positively skewed and the simpler linear mixed model failed to fit the data without violation of its assumptions.

In the results section, mean MEP sizes (MEPabs and MEP%) estimated with the models are reported along with standard errors, 95% confidence intervals and relevant hypothesis tests with significance level set at 0.05. No adjustments were applied for multiple comparisons as it reduces type‐I error at the cost of increased type‐II error.^24^

## Results

Twelve participants were screened for the trial. Three of which them were excluded; two due to the absence of a resting‐state MEP from the paretic TA muscle, and one could not tolerate TMS. The remaining nine participants completed all four interventions and data from these were analyzed.

### BCI system performance

There were no statistically significant differences in performance metrices between the asynchronous and cue‐based asynchronous‐BCI systems (*p* > 0.05) except in the number of FP/min, which was lower for the cue‐based asynchronous system compared to the asynchronous system (average difference = 0.40 FP/min and 8% FP's/TP's). Means and standard deviations of the TPR, FP/min, time duration of the testing session, false positive/true positives (%), and the number of movements attempts during the testing session are presented in Table 2.

**Table 2:** Performance metrices (mean ± SD) of the cue‐based asynchronous and asynchronous BCIs.

Performance metric	Cue‐based asynchronous BCI	Asynchronous BCI	*p*‐value (*t*‐test)
True positive rate	84.88 ± 5.34	82.68 ± 11.67	0.50
False positives/minute	0.52 ± 0.17	0.99 ± 0.60	0.03*
false positive/true positives (%)	10.67 ± 3.16	18.44 ± 11.08	0.03*
Intervention length (minutes)	10.51 ± 0.78	9.39 ± 2.65	0.19
Movement attempts	59.11 ± 3.68	61.67 ± 9.55	0.37
True positive rate	84.88 ± 5.34	82.68 ± 11.67	0.50

* *p* <0.05.

### MEP size – absolute units

**Figure 2:**
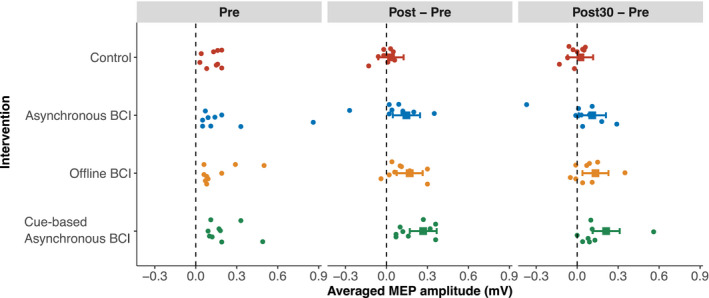
Averaged peak‐peak MEP amplitudes in millivolts of individual participants. The pre‐intervention MEP amplitudes are subtracted from the post‐ and post30‐intervention MEP values to highlight the pre‐ to post‐ and post30‐intervention change. The means and 95% confidence intervals estimated from the statistical model are also presented with squares and interval lines.

Compared to the control intervention, all BCI interventions increased the MEP amplitude at post‐ and post30‐intervention time points with respect to pre‐intervention MEP amplitudes. The means estimated from the statistical model for the averaged peak‐peak MEP amplitude for the four interventions at post‐ and post30‐ are given in Table 3 and also superimposed on averaged peak‐peak MEP amplitudes of individual participant data in Figure 2. These means were estimated after adjusting for the pre‐intervention MEP amplitude differences across the participants and considering the pre‐intervention value as 0 mV. The corresponding hypothesis tests show that all BCI modes resulted in statistically significant increases in MEP amplitudes immediately post‐intervention and 30‐min post‐intervention, whereas the control condition did not affect MEP amplitude.

**Table 3:** Estimated averaged peak‐peak MEP amplitude from the statistical model.

Session	Time	Estimate ± S.E. (mV), 95% C.I. [lower, upper]	H0: Estimate = 0 mV *t*‐value [df], *p*‐value
Control	post‐	0.033 ± 0.046, [−0.060, 0.126]	0.707 [49.3], 0.483
	post30‐	0.024 ± 0.046, [−0.069, 0.117]	0.515 [49.3], 0.609
Asynchronous BCI	post‐	0.146 ± 0.050, [0.046, 0.246]	2.940 [51.1], 0.0049*
	post30‐	0.111 ± 0.050, [0.010, 0.211]	2.225 [51.1], 0.0305*
Offline BCI	post‐	0.170 ± 0.047, [0.075, 0.265]	3.588 [49.9], 0.0008*
	post30‐	0.135 ± 0.047, [0.039, 0.230]	2.838 [49.9], 0.0066*
Cue‐based asynchronous BCI	post‐	0.269 ± 0.049, [0.170, 0.367]	5.471 [50.8], <0.0001*
	post30‐	0.212 ± 0.049, [0.114, 0.311]	4.318 [50.8], 0.0001*

S.E., standard error; C.I., confidence interval, H0, null hypothesis; df, degrees of freedom. The post‐ and post30‐intervention MEP amplitudes are adjusted with pre‐intervention MEP amplitude set to 0 mV.

* *p* <0.05.

The mean MEP amplitudes from the four interventions and comparison to each other are presented in Table 4. These tests suggest that immediately post‐intervention, all three BCI modes induced a larger increase in MEP amplitude compared to the control condition. In contrast, at post30‐intervention, only the cue‐based asynchronous‐BCI had larger MEP amplitudes compared to the control condition. When comparing the BCI conditions, the cue‐based asynchronous‐BCI resulted in larger increase in MEP amplitude immediately post‐intervention compared to the asynchronous‐BCI. There were no other statistically significant differences. Besides the significance tests, the trends in effect sizes favored the following hypothesis: cue‐based asynchronous‐BCI > offline‐BCI = asynchronous‐BCI > control, that is, all BCI interventions performed better than the control intervention; cue‐based asynchronous‐BCI performed the best whereas the other two BCI modes had a similar performance.

**Table 4:** Comparison of interventions based on estimated averaged peak‐peak MEP amplitudes.

Comparison	Time	Contrast ± S.E. (mV), 95% CI [lower, upper]	H0: Contrast, 0 mV *t*‐value[df], *p*‐value
Asynchronous BCI – control	post‐	0.114 ± 0.056, [0.001, 0.227]	2.013 [55.5], 0.049*
	post30‐	0.087 ± 0.056, [−0.026, 0.200]	1.540 [55.5], 0.129
Offline BCI – control	post‐	0.137 ± 0.056, [0.026, 0.249]	2.464 [55.1], 0.017*
	post30‐	0.111 ± 0.056, [−0.001, 0.222,]	1.985 [55.1], 0.052
Cue‐based asynchronous BCI – control	post‐	0.236 ± 0.056, [0.124, 0.349]	4.203 [55.4], 0.0001*
	post30‐	0.188 ± 0.056, [0.076, 0.301]	3.352 [55.4], 0.001*
Offline BCI – asynchronous BCI	post‐	0.024 ± 0.056, [−0.088, 0.136]	0.426 [55.2], 0.672
	post30‐	0.024 ± 0.056, [−0.088, 0.136]	0.426 [55.2], 0.672
Cue‐based asynchronous BCI – asynchronous BCI	post‐	0.123 ± 0.056, [0.011, 0.234]	2.203 [55.0], 0.032*
	post30‐	0.102 ± 0.056, [−0.010, 0.213]	1.824 [55.0], 0.074
Cue‐based asynchronous BCI – Offline BCI	post‐	0.099 ± 0.056, [−0.013, 0.211]	1.771 [55.1], 0.082
	post30‐	0.078 ± 0.056, [−0.034, 0.190]	1.392 [55.1], 0.169

S.E., standard error; C.I., confidence interval, H0, null hypothesis; df, degrees of freedom.

* *p* <0.05.

### MEP size – relative units

Similar to absolute MEP amplitude, relative MEP results demonstrated that the control condition did not increase MEP amplitudes, whereas all BCI modes increased MEP amplitudes at post‐ and post30‐intervention timepoints. No statistically significant differences were detected between the interventions, except for the cue‐based asynchronous‐BCI versus the control at post‐intervention, where the cue‐based asynchronous‐BCI MEP amplitudes were 109.4% higher compared to the control condition. The means estimated for the percentage change in averaged peak‐peak MEP amplitudes during the four sessions and their comparisons are presented in Tables 5 and 6, respectively.

**Table 5:** Estimated MEP percentage change in averaged peak‐peak MEP amplitude from the statistical model.

Intervention	Time	Estimate ± S.E. (%), 95% C.I. [lower, upper]	H0: Estimate = 0% *z*‐value, *p*‐value
Control	post‐	31.5 ± 31.3, [−29.8, 92.8]	1.006, 0.314
	post30‐	28.8 ± 31.3, [−32.5, 90.1]	0.922, 0.357
Asynchronous BCI	post‐	115.4 ± 33.3, [50.2, 180.7]	3.466, 0.0005*
	post30‐	106.6 ± 33.3, [41.4, 171.9]	3.203, 0.001*
Offline BCI	post‐	114.9 ± 31.9, [52.3, 177.5]	3.597, 0.0003*
	post30‐	84.8 ± 31.9, [22.2, 147.4]	2.655, 0.008*
Cue‐based asynchronous BCI	post‐	140.8 ± 32.9, [76.3, 205.4]	4.275, <0.0001*
	post30‐	111.1 ± 32.9, [46.5, 175.7]	3.373, 0.0007*

S.E., standard error; C.I., confidence interval, H0, null hypothesis; df, degrees of freedom. The post‐ and post30‐intervention MEP amplitudes are adjusted with pre‐intervention MEP amplitude set to 0 mV.

* *p* <0.05.

**Table 6:** Comparison of interventions based on the estimated percentage change in averaged peak‐peak MEP amplitudes.

Comparison	Time	Contrast ± S.E. (%), 95% CI [lower, upper]	H0: Contrast = 0% *z*‐value, *p*‐value
Asynchronous BCI – control	post‐	83.9 ± 42.9, [−0.2, 168.1]	1.955, 0.051
	post30‐	77.8 ± 42.9, [−6.3, 162.0]	1.812, 0.070
Offline BCI – control	post‐	83.4 ± 42.6, [−0.1, 166.9]	1.958, 0.050
	post30‐	56.0 ± 42.6, [−27.5, 139.5]	1.314, 0.190
Cue‐based asynchronous BCI – control	post‐	109.4 ± 42.8, [25.4, 193.3]	2.553, 0.011*
	post30‐	82.3 ± 42.8, [−1.7, 166.2]	1.921, 0.055
Offline BCI – asynchronous BCI	post‐	−0.5 ± 42.7, [−84.2, 83.2]	−0.012, 0.990
	post30‐	−21.9 ± 42.7, [−105.6, 61.8]	−0.512, 0.609
Cue‐based asynchronous BCI – asynchronous BCI	post‐	25.4 ± 42.6, [−58.0, 108.9]	0.598, 0.543
	post30‐	4.5 ± 42.6, [−79.0, 87.9]	0.105, 0.917
Cue‐based asynchronous BCI – Offline BCI	post‐	26.0 ± 42.6, [−57.6, 109.5]	0.609, 0.543
	post30‐	26.3 ± 42.6, [−57.3, 109.9]	0.617, 0.537

S.E., standard error; C.I., confidence interval, H0, null hypothesis; df, degrees of freedom.

* *p* <0.05.

## Discussion

### BCI efficacy

This study aimed to determine the effect of the proposed cue‐based asynchronous‐BCI mode compared to the two traditional BCI systems (asynchronous and offline) and a control intervention on corticomotor excitability in people with stroke. The findings demonstrated that all three BCI interventions significantly increased corticomotor excitability (peak‐to‐peak MEP amplitude) immediately post‐intervention, and this increase was sustained for 30 min. Conversely, the control condition did not affect corticomotor excitability. Moreover, the novel cue‐based asynchronous‐BCI produced a significantly greater increase in absolute MEP size immediately post‐intervention compared to the asynchronous system. Overall, the effect size estimates suggested that the proposed cue‐based asynchronous‐BCI was superior to all other interventions for inducing plasticity. The offline and asynchronous‐BCIs had similar performance but were better than the control intervention.

The relative data showed that the cue‐based asynchronous‐BCI intervention increased MEP amplitude by >140%, compared to 115% for the other two BCI modes. The magnitude of this increase can be compared with other studies that have reported enhanced post‐stroke corticomotor excitability following other single‐ or multi‐session neuromodulatory interventions. A single session of anodal transcranial direct current stimulation (tDCS) over the ipsilesional motor cortex has produced significant increases in upper limb MEP amplitudes of 68%^25^ and 21%,^26^ with a moderate effect size (0.59).^27^ Application of a single session of inhibitory paired associative stimulation to the contralesional hemisphere and inactive non‐affected target muscle during treadmill walking increased relative MEP amplitudes of the affected TA muscle by 20–34%.^28^, ^29^ A recent systematic review and meta‐analysis evaluating the effect of multi‐session repetitive TMS (rTMS) found higher ipsilesional lower limb MEP amplitudes (standardized mean difference = 1.13).^30^ MEP amplitudes of the affected rectus femoris muscle have been reported to increase by 44% following repeated contralesional rTMS stimulation and task‐specific training.^31^ Whereas the application of repeated anodal tDCS over the ipsilesional lower limb motor cortex combined with usual physiotherapy care increased the MEP amplitude of the affected TA muscle by 279%.^32^ As the increase in MEP amplitude noted in our study surpasses the changes seen following other single session^25^, ^26^, ^28^, ^29^, ^30^ and even multi‐session^31^ neuromodulatory interventions, this highlights the potential of the cue‐based asynchronous‐BCI intervention to modulate post‐stroke lower limb motor cortex excitability. Future research needs to establish whether the post‐BCI MEP increase is associated with clinically meaningful motor function changes in people with stroke by utilizing a single or a multi‐session study design.

Measures of corticomotor excitability are prone to high inter‐subject variability.^33^ This is demonstrated in Figure 2 by the spread of the data points. The natural variability at baseline is further intensified post‐intervention due to the variable response to neuromodulatory interventions.^34^, ^35^, ^36^, ^37^ Response to neuromodulation is known to be influenced by multiple factors (see^33^, ^38^ for a review). Attention may have differed between the asynchronous‐BCI, where the participant‐led the movement initiation, and the cue‐based asynchronous‐BCI and offline‐BCI that were visually cued may have maintained attention more effectively.

### Mechanisms of neural plasticity

The observed changes in corticospinal excitability could be mediated through long‐term potentiation‐like mechanisms, as proposed in previous studies.^14^, ^19^ The criteria for long‐term potentiation‐like plasticity are associativity, rapid onset, lasting effects (exceeding the intervention period), and specificity.^39^ The post‐intervention increases in MEP amplitude observed in this study fulfill the rapid onset and lasting effects criteria. This study did not investigate the specificity of effects, however, previous research investigating the offline‐BCI protocol has confirmed that the effects are specific to the stimulated muscle.^19^ All three BCI protocols somewhat fulfill this criterion in terms of associativity by pairing movement intentions with timely somatosensory input from the peripheral nerve electrical stimulation. However, the accuracy of this timing is likely to have differed between the three protocols. There will likely be some error in the offline‐BCI protocol, as the electrical stimulation's timing is based entirely on the pre‐recorded averaged MRCP rather than real‐time EEG.

In contrast, the two asynchronous systems monitor real‐time EEG to determine the timing of the electrical stimulation. Both asynchronous systems provided equivalent true‐positive‐rates for detection of the MRCP, and both systems delivered 50 correct stimulations as per protocol. However, the more efficacious BCI protocol (cue‐based asynchronous) had significantly lower false positives per 100 true positives. This might suggest that the incorrect delivery of electrical stimulation when a participant is not attempting to move (i.e., when a false positive has been detected) may be detrimental to the development of associative plasticity. Fewer mistimed pairings achieved by the cued‐based asynchronous‐BCI may explain its higher efficacy. This may occur throughout the central or peripheral nervous system in terms of the origin of neural plasticity. However, previous studies have found no change in stretch reflex excitability, suggesting the location of changes is supraspinal.^12^, ^13^, ^19^

### Clinical translation

The proposed cue‐based asynchronous‐BCI had the best performance in this study, but an additional advantage of this system is its potential usability at home by people with stroke. One aspect of usability is the setup process. In comparison to the offline BCI, the cue‐based asynchronous‐BCI detects the onset of the MRCP rather than needing the intervention parameters to be entered, thus, making the set‐up more straightforward. Another aspect of usability is the level of cognition, attention and motivation required to participate successfully. The cue‐based asynchronous‐BCI provides visual cues that outline the exact steps required to the patient, making it potentially more engaging and simpler to understand than the asynchronous‐BCI in which the patient needs to self‐initiate the movement. Thus, some aspects of the cue‐based asynchronous‐BCI might offer improved usability. It is easier to set up and likely put a less cognitive load on the patients. Also, it is suggested in previous literature that the use of BCI is a skill.^40^ Thus it is possible that repeated sessions would lead to better learning and performance.

The BCIs in this study utilized peripheral electrical stimulation paired with the MRCP. However, recent studies have indicated that neural plasticity can also be induced by pairing robot‐assisted passive movement with the MRCP.^10^, ^41^ This allows for a greater set of choices for the intervention and extends the viability of the treatment to patients who cannot receive electrical stimulation due to contraindications or discomfort. Further research should investigate other modes of delivering afferent stimulation within this BCI paradigm, considering both efficacy and usability.

In healthy subjects, the BCI‐triggered system has been substituted by an EMG‐detector that triggers electrical stimulation and results in equivalent effects on neural plasticity.^14^ This EMG‐triggered system could also be useful in patients with stroke. However, this would require EMG activity. It is likely that more severely impaired stroke patients may not have sufficient EMG to drive the EMG‐triggered system, and therefore would require the EEG‐based BCI. Those patients could initially start the rehabilitation with a BCI, and with improvements in EMG activity, they could be transferred to EMG‐based rehabilitation.^42^ An EMG system would have advantages in terms of usability due to its more convenient set‐up and lower cost.

As in previous BCI studies,^10^, ^12^, ^13^, ^14^, ^20^ this study used 50 pairings of movement intention with somatosensory stimulation to induce changes in neural plasticity. This protocol has previously resulted in improvements in lower limb impairment for individuals with stroke.^11^, ^43^ However, the optimal dose (amount) of pairings within the BCI protocol is not known. The induction of neural plasticity or its retention may increase with greater repetitions, as reported for lower limb paired associative stimulation (see^38^ for a recent review). Thus, further research should investigate dose–response relationships to optimize the efficacy of this intervention and improve its potential for implementation into clinical practice where the BCI system will be used in the long term. Another avenue for future work is to look at the use of the BCI as an adjunct to standard rehabilitation. The duration of its excitatory effects has been reported to last for at least 60 min.^44^ Therefore, the BCI could be used to prime the nervous system, for example before a physiotherapy session.

### Limitations

The results must be interpreted with caution as it is pilot trial, where we have implemented the combination of the cue‐based and self‐paced BCI approaches. While MEP size increased post‐intervention, which indicates that neural plasticity was induced, this does not suggest the occurrence of any long‐term changes in motor learning. A more extended training period would likely be needed to induce changes in motor learning, and these could only be confirmed by recording retained improvements in motor function. However, the immediate increase in neural plasticity indicates that the BCI training could be a useful stroke rehabilitation tool.^9^ This is supported by other BCI studies showing a training effect in people with stroke.^11^, ^43^ Moreover, a limited sample was included, and it is difficult to generalize the results to the broader stroke population, which is very heterogeneous.

The eligibility criteria specified that an MEP should be elicited in response to TMS, which indicates all participants had a certain level of corticomotor excitability at baseline. If plasticity‐inducing BCIs, such as those used in this study, will be tested with more severely impaired patients, measures other than TMS are needed.^45^, ^46^ Potential electrophysiological parameters could be EEG–EMG coherence or connectivity to measure transient/lasting brain changes or functional measures such as muscle strength and neuromuscular fatigue.^47^

## Conclusion

The proposed cue‐based asynchronous‐BCI and the two traditional BCI modes (asynchronous and offline) resulted in increased corticomotor excitability compared to the control condition in people with chronic stroke. Notably, the proposed cue‐based asynchronous‐BCI outperformed both the asynchronous and offline BCIs, which had a comparable performance. The lower number of false positives in MRCP detections may explain the greater efficacy of the cue‐based asynchronous‐BCI. The results of this pilot study support the potential use of the BCI as a tool to increase neural plasticity and functional recovery in people with stroke. Further research is needed to optimize the efficacy, modes of delivery, and usability of this approach for stroke rehabilitation.

## Authors’ Contributions

IKN, MSN, UR, HH, NS, DT, DF, and MJ contributed to the conception and design of the study. IKN, MSN, UR, and IA, contributed to the acquisition of data. IKN, MSN, UR, NK, SO, GA, HH, NS, DT, DF, and MJ contributed to the analysis and interpretation of data. IKN, MSN, and UR contributed to the drafting and revision of the manuscript. All authors have read and agreed to the published version of the manuscript.

## Conflicts of Interest

Nothing to report.

## Data Availability

The data that support the findings of this study are available from the corresponding author, upon reasonable request.
